# Exercise prehabilitation for brain health and sensory–cognitive function: Mechanistic insights into brain–body interactions

**DOI:** 10.1113/EP092907

**Published:** 2025-09-23

**Authors:** Helen E. Nuttall, Daren Subar, Christopher J. Gaffney

**Affiliations:** ^1^ Department of Psychology Lancaster University Bailrigg UK; ^2^ East Lancashire Hospitals Trust Blackburn UK; ^3^ Lancaster Medical School Lancaster University Bailrigg UK

**Keywords:** brain, cognition, exercise physiology, neuroscience, prehabilitation, sensory input

## Abstract

This mini review article presents a novel hypothesis for extending exercise prehabilitation into the domains of brain health and cognition. Whilst prehabilitation has been gaining popularity in cancer treatment, conferring significant positive benefits to numerous physiological indicators, including post‐operative infection and hospital length of stay, the benefits to patients' brain health and cognition are underexplored. There lies a timely and exciting opportunity in expanding prehabilitation to target neural and sensory dysfunction in cancer patients. We review data from healthy adults on the benefits of exercise to brain structure and function, and cognitive and sensory domains. Finally, we review how exercise could improve brain health, as well as sensory systems of the body, which are often negatively affected by cancer and associated treatment. We also outline further physiological contexts outside of cancer in which prehabilitation could also benefit brain health. Through this synthesis, we seek to inspire novel research into prehabilitation and brain health to further improve patient health and wellbeing.

## Abstract (highlights)

- **What is the topic of this review?**
  This review focuses on how exercise prehabilitation in cancer care may also confer positive benefits to brain health and cognitive function, in addition to the domains of physical health that are already being explored.
- **What advances does it highlight?**
  It highlights that consideration of patients’ brain health is overlooked, yet there is a significant and timely opportunity to expand exercise prehabilitation to measures of brain health, cognitive and sensory function, and the mechanisms behind possible benefits.

## 1 EXERCISE PREHABILITATION AND POST‐SURGICAL HEALTH

An exciting area in physiology research is to use exercise as prehabilitation. In this article, we present the idea that the benefits of prehabilitation have not yet been fully realised. Specifically, a novel investigation of how prehabilitation is also affecting patients’ brain health and cognition is an underexplored avenue that holds great potential. Whereas rehabilitation takes place after surgery, prehabilitation takes place before surgery in order to maximise health before surgery and optimise surgical outcomes (Lambert et al., 2024). Surgical outcomes so far have been largely focused on changes to physical health, overlooking any cognitive or brain‐based changes. Like running a marathon, surgery is a huge stressor on the human body, and exercise training in the form of prehabilitation prepares the body for this challenge. Prehabilitation has been shown to improve cardiorespiratory fitness before cancer surgery, which improves functional capacity, reduces post‐operative complications, and reduces hospital length of stay (Lambert et al., 2021), thus offering patient‐centred and wider economic benefits. Currently, no measures of cognition or brain health are routinely integrated into prehabilitation evaluation.

The two most frequently studied forms of prehabilitation are exercise and nutritional interventions. It has been shown that pre‐operative exercise increases fitness before an operation, with several studies observing improvements in cardiopulmonary exercise test variables (${\dot{V}}_{O_{2}\max}$ and anaerobic threshold) and functional capacity after supervised and unsupervised pre‐operative exercise programmes. Data also indicate that greater fitness before surgery is associated with faster recovery, following, for example, major abdominal surgery (Carli et al., 2010). Furthermore, benefits from prehabilitation include reduced hospital length of stay (Lambert et al., 2021) and reduced complications (Bousquet‐Dion et al., 2018). The majority of cancers in the UK are diagnosed in the older adult population (aged 65 years and above). A multitude of different prehabilitation intervention types are used, incorporating aerobic and resistance exercise, which are tailored to the maximum capabilities of the patients, with multimodal approaches gaining favour (Lambert et al., 2021). The most effective form of prehabilitation, however, has yet to be identified. As such, exercise prehabilitation has been established as a safe and effective intervention for older adults preparing for surgery.

Prehabilitation programmes are typically only 2–6 weeks long to allow patients to undergo surgery at the earliest opportunity. There is evidence of improvement in trials of 2–3 weeks (Liu et al., 2020). Longer programmes of 4 weeks in cardiac, thoracic, GI and major abdominal surgery have shown improved functional capacity and reduced post‐operative complications (Yau et al., 2025). Programmes of 6 weeks have shown improvements in ${\dot{V}}_{O_{2}\max}$ and postoperative morbidly (Michel et al., 2022). The dose of exercise required to improve brain function in a chemotherapy cohort is not known, which is why this review is timely and significant. Similar to prehabilitation exercise interventions, research in the area of high intensity interval training (HIIT) (Little et al., 2011) has shown that as little as six sessions of HIIT (10 × 60‐s cycling bouts eliciting ∼90% maximal heart rate, interspersed with 60 s rest) over 2 weeks can elicit metabolic improvements in physiology, specifically increases in mitochondrial function.

## 2 EXERCISE AND BRAIN STRUCTURE

What is even more exciting about exercise prehabilitation is that we hypothesise that it has more to give. In this piece, we share our view that the full benefits of prehabilitation have not yet been fully realised, and this timely, emerging area has great potential to impact physiology in several different directions. Specifically, there exists great benefit to the brain following prehabilitation, which could maximise the impact of prehabilitation on patient outcomes and mitigate cognitive dysfunction. Whilst using prehabilitation to specifically optimise ‘brain fitness’ prior to a stressor to the brain is a relatively new idea, the general benefits of exercise to brain health are well‐established.

Chronic exercise increases growth factors in the blood, which collectively improve neuroplasticity. These include brain‐derived neurotrophic factor (BDNF) and vascular endothelial growth factor (VEGF). Data indicate that BDNF in healthy older adults can be upregulated by moderate intensity endurance training (Liang et al., 2023). BDNF is a neurotrophin that plays an important role in synaptic plasticity, neuronal survival, differentiation and neuronal growth. Moreover, BDNF is strongly linked to cognitive function. Exercise‐induced neuroplasticity can be evaluated by observing changes in cerebral grey matter, measured through magnetic resonance imaging. Increased grey matter indicates positive neural changes, and offers important insights into how exercise influences brain structure. Research in mice has demonstrated that voluntary aerobic training enhances hippocampal neurogenesis, long‐term potentiation and learning (Boa Sorte Silva et al., 2024). These effects likely result from exercise‐induced upregulation of neurotrophic factors, such as BDNF.

In humans, aerobic training in cognitively normal older adults has also been shown to increase hippocampal volume, which indeed correlates with increased BDNF (Erickson et al., 2011). Individuals with cognitive decline may be more sensitive to exercise‐induced increases in hippocampal neuroplasticity relative to cognitively healthy individuals. Nonetheless, in cognitively healthy individuals, the effects of aerobic training are numerous, with increases in whole‐brain and regional (e.g., frontal, temporal and cingulate) grey matter volumes (Colcombe et al., 2006). A range of neurobiological adaptations may underpin such grey matter increases, including synaptogenesis, axonal sprouting, dendritic branching/arborisation, and angiogenesis. Therefore, the positive effects of exercise‐induced neuroplasticity in clinical and non‐clinical groups are wide‐ranging, with evidence of whole‐brain and regional increases in grey matter, likely catalysed by upregulation of neurotrophins such as BDNF. This is in addition to increases in white matter volume, greater connectivity within the nervous system, and the formation of new blood vessels that increase cerebral blood flow. Thus, exercise improves brain health and function through a variety of physiological mechanisms, in both clinical and non‐clinical groups.

Whilst there are data to show that exercise can improve markers of neuroplasticity (El‐Sayes et al., 2019; Rich et al., 2017), the ‘dose’ of exercise required to impact brain structure in a chemotherapy cohort is not known, which is why this review is timely and significant as it presents another exciting area for exploration. Meta‐analysis (Szuhany et al., 2014) indicates reliable evidence that both acute and regular exercise have a significant impact on BDNF levels in healthy adults. Evidence suggests that a single session of exercise increases BDNF levels (exercise types were predominantly aerobic and included a range of types, including rowing, running, and cycling, at a range of different intensities from moderate through to exhaustion; Szuhany et al., 2014). Moreover, regular exercise (ranging from 2 to 24 weeks, and again across a variety of different exercise types and intensities, predominantly aerobic and moderate intensity; Szuhany et al., 2014) intensifies the extent of acute effects, increasing BDNF responsivity, relative to those completing acute exercise alone. Programmes of regular exercise were also found to impact resting BDNF levels (Szuhany et al., 2014). These data suggest that each episode of exercise could be considered a ‘dose’ of BDNF activity, and over time, the size of the ‘dose’ can be enhanced by regular exercise. This means that as well as demonstrating subtle increases in resting BDNF, individuals engaged in a regular exercise programme can episodically ‘top‐up’ their BDNF doses. Aerobic training appears to most consistently modulate BDNF, but limited evidence directly comparing effects of aerobic versus strength training on BDNF certainly presents an area for future study.

## 3 EXERCISE AND COGNITIVE FUNCTION

Exercise‐induced changes to brain structure are also linked to changes in brain function, specifically cognition. Domains of executive function and memory have been extensively documented in prior investigations into the effect of exercise on cognition because such cognitive domains are particularly sensitive to age‐related declines and neurodegenerative processes. Following exercise training, data indicate that there is consistent improvement in executive functioning. Executive function skills help us plan, focus attention and switch tasks. We rely on executive function skills extensively in day‐to‐day life; for example, they enable us to cook dinner safely whilst also listening to how our spouse's day was. Improvements in executive function have important implications for healthy ageing given the central role of executive functions in behaviours relevant to functional independence.

Exercise‐induced improvements in executive function are likely brought about by neuroplastic adaptations in the prefrontal cortex (Friedman & Robbins, 2021), which subserves executive function. Such skills deteriorate with healthy ageing, and deterioration is accelerated in individuals with Alzheimer's disease. Changes in memory function, particularly episodic memory that is dependent upon the hippocampus, are common in healthy ageing, as well as in pathological ageing (e.g., Alzheimer's disease). Research suggests that exercise has a positive effect on episodic memory, although effects are not consistently replicated across meta‐analyses (Young et al., 2015). One reason for this disparity may relate to variations in how memory is tested and operationalised in different studies. Therefore, findings of exercise‐driven neuroplasticity in the hippocampus in the context of healthy ageing suggest this area as ripe for further research and development to better understand the precise nature of exercise‐induced neurobiological adaptations in memory, and how this presents behaviourally.

## 4 EXERCISE PREHABILITATION TO AMELIORATE CHEMOTHERAPY‐INDUCED COGNITIVE DYSFUNCTION

We hypothesise that exercise prehabilitation to target neurobiological adaptations in prefrontal cortex for neuroprotection and optimisation of brain fitness prior to chemotherapy treatment holds huge promise in reducing cognitive dysfunction following chemotherapy. Chemotherapy‐induced neurotoxicity may affect any neuron in the body, both directly (direct interaction with the cell body and neurites) and indirectly (due to glial damage, inflammation and other mechanisms) and thus may cause many different symptoms affecting the quality of life of patients undergoing anti‐cancer treatment. Neurotoxicity affecting the brain results in ‘chemo brain’, defined by the National Cancer Institute as:

*A term commonly used to describe thinking and memory problems that a patient with cancer may have before, during, or after cancer treatment. Signs and symptoms of chemo brain include disorganized behaviour or thinking, confusion, memory loss, and trouble concentrating, paying attention, learning, and making decisions*.

It is uncertain how many people with cancer experience cognitive dysfunction following cancer treatment. Research indicates that up to 75% of people experience cognitive changes during treatment, and up to 35% have symptoms after treatment (Cancer Research UK, 2025). With over 2 million cancer survivors in the UK, these data are staggering and indicate that interventions and support for cognitive dysfunction are of paramount importance. In breast cancer, for example, data indicate that survivors who have been treated with adjuvant chemotherapy experience long‐term cognitive deficits that significantly reduce quality of life (Kesler et al., 2011). Such individuals show altered brain structure and function compared with patients who were not treated with chemotherapy, suggesting a pattern of diffuse brain injury that may underpin cognitive deficits.

In particular, the most common cognitive dysfunction experienced among breast cancer survivors is impairment in abilities associated with executive function, such as problems with working memory, cognitive flexibility, multitasking, planning and attention (Yamada et al., 2010). In line with this, structural and functional brain imaging has indicated changes to the prefrontal cortex, which subserves executive function (Kesler et al., 2011). Whilst priority should be on cancer survival, the effects of chemotherapy treatment on quality of life are incredibly concerning and extend disease‐related disability (Boykoff et al., 2009). Indeed, a key focus should be on extending patients’ healthspan in line with lifespan, supporting workplace productivity and healthy ageing. With 80% of breast cancers occurring in women aged 50 years and above (Breast Cancer Now, 2025), combined with increasing retirement age, cognitive dysfunction in breast cancer patients will have significant negative consequences for workplace performance and daily living (Boykoff et al., 2009), which in turn will negatively impact psychological wellbeing. Many women show continued neurobiological and cognitive deficits at 10–20 years follow‐up post‐treatment.

Given the known benefits of exercise to brain health and cognition outlined above, exercise prehabilitation presents an ideal intervention to ameliorate cognitive dysfunction following chemotherapy and holds significant promise in improving long‐term quality of life for cancer survivors. This is not just in the context of breast cancer, and will apply to chemotherapy and radiotherapy treatments associated with an extensive array of cancer types. As such, exercise prehabilitation should be a priority for cancer patients, and markers of cognitive status included in standard care to ensure timely identification of impairment and targeted support.

## 5 EXERCISE PREHABILITATION TO AMELIORATE CHEMOTHERAPY‐INDUCED SENSORY DYSFUNCTION

As well as central dysfunction observed via brain structure and cognitive processing, chemotherapy can also cause dysfunction in sensory systems, also linked to inflammation and neurotoxicity. As such, we also hypothesise that exercise prehabilitation may reduce, for example, the ototoxic effects of chemotherapy on the hearing system. Indeed, platinum‐based chemotherapy drugs are ototoxic, meaning they cause damage to the hair cells in the ear leading to hearing loss (Baguley & Prayuenyong, 2020). The most ototoxic chemotherapy drug is cisplatin, which is used to treat testicular, ovarian, lung, bladder, head and neck, oesophageal, cervical, and stomach cancers. Whilst these cancer types can be aggressive, and may be managed rather than ‘cured’, they are indeed ones where there may be most benefit from prehabilitation, as the negative effects of treatment are more sustained.

Platinum‐based chemotherapy agents cause oxidative stress and cell death in cochlear cells in the ear, which leads to permanent damage in the hearing system. Research indicates that ototoxicity‐induced hearing loss is linked to excessive generation of reactive oxygen species (ROS) in cells of the cochlea. Such excessive generation of ROS could lead to cochlear inflammation, suggesting the use of anti‐inflammatory agents for treatment of hearing loss. However, it is also well‐established that exercise can reduce the whole‐body inflammatory response by, for example, reducing pro‐inflammatory cytokines, such as IL‐1β and IL‐18 (Ding & Xu, 2021). The mechanisms of exercise's protective effects are somewhat elusive but may be anti‐inflammatory (IL‐6) derived. IL‐6 production from leukocytes drives tumorigenesis, yet IL‐6 production from skeletal muscle during exercise improves insulin sensitivity, promotes anti‐inflammatory cytokine production, and prevents DNA damage (Orange et al., 2023), collectively offering an ‘anti‐cancer’ effect.

The pro‐inflammatory activity resulting from platinum‐based chemotherapy may lead to ototoxic cell death of cochlear hair cells, causing hearing loss. But critically, we hypothesise that the anti‐inflammatory effects of exercise prehabilitation prior to and during chemotherapy could blunt the extent of sensory dysfunction arising from inflammation, that is, reducing ototoxicity and supporting hearing function during cancer treatment, which has never been tested. This hypothesis could also be extended to chemotherapy‐induced dysfunction in other sensory systems affected by inflammation. As such, our hypotheses have wide‐ranging possibilities for improving quality of life in cancer patients. We assert that exercise prehabilitation could catalyse changes that not only improve post‐surgical outcomes, but could also lead to a cascade of positive implications for sensory and cognitive dysfunction caused by chemotherapy, and by extension, radiotherapy.

## 6 FUTURE DIRECTIONS

There are many types of cancer where we hypothesise prehabilitation could benefit cognition and brain health. Cancers treated with collections of drugs with documented side effects on the brain, for example patients treated with platinums, anthracyclines, taxanes, antimetabolites, and hormonal therapies, would also be good avenues to explore therapeutic benefit. Blood cancer, such as chronic leukaemia and multiple myelomas, could potentially benefit. Similarly, low‐grade non‐Hodgkin lymphomas or metastatic cancers including breast, prostate, ovarian and colorectal cancers could benefit. There are some cancers where prehabilitation may have little benefit, and typically these would be where the performance status of a patient is so low that it is difficult to conduct any exercise. However, these patients could still potentially benefit from bed‐focused interventions, such as the use of passive exercise.

Further, whilst we have focused on the extensive physiological benefits of exercise prehabilitation on neural, cognitive, and sensory function to improve cancer patients’ future quality of life, the promise of prehabilitation does not lie solely within the field of cancer research. Any clinical (or non‐clinical) context where cognitive function may be disrupted due to pharmaceutical side effects, such as hormone therapy, anticholinergics or antidepressant medications, could theoretically benefit from exercise prehabilitation in advance of starting such medication. Similarly, instances where neuroplasticity is required to support brain repair or learning in response to a medical implant, such as a prosthetic limb or cochlear implant, could theoretically benefit from exercise prehabilitation. These are just a small number of additional circumstances where exercise prehabilitation could be implemented before a known stressor to the brain. In this way, prehabilitation could be used to optimise brain fitness and maximise the neural environment for cognitive function and/or novel learning, in order for the brain to respond more effectively when presented with such brain stressors. In our opinion, future research should explore much more widely the benefits of exercise prehabilitation to the brain and cognitive function, to fully unlock the utility of exercise prehabilitation for healthier futures.

## AUTHOR CONTRIBUTIONS

H.E.N. contributed to conceptualisation, funding acquisition, investigation, resources, writing—original draft, and writing—review and editing. D.S. contributed to funding acquisition, writing—reviewing and editing. C.J.G. contributed to conceptualisation, funding acquisition, resources, and writing—review and editing. All authors have read and approved the final version of this manuscript and agree to be accountable for all aspects of the work in ensuring that questions related to the accuracy or integrity of any part of the work are appropriately investigated and resolved. All persons designated as authors qualify for authorship, and all those who qualify for authorship are listed.

## CONFLICT OF INTEREST

None declared.
